# SUPERCONDUCTIVITY: CHALLENGE FOR THE FUTURE Federal Conference on Commercial Applications of Superconductivity, Washington, DC, July 28–29, 1987

**DOI:** 10.6028/jres.092.041

**Published:** 1987-12-01

**Authors:** Robert A. Kamper, Alan F. Clark

**Affiliations:** Electromagnetic Technology Division, National Bureau of Standards, Boulder, CO 80303

President Reagan outlined a new Superconductivity Initiative at the Federal Conference on Commercial Applications of Superconductivity, Washington, DC, July 28–29, 1987. The primary purpose of this conference was to stimulate commercial enterprise in high-temperature superconductor technology. Various sessions summarized the scientific facts so far as they are known, described the existing superconductor technology and attempted to draw lessons from it, speculated on the possible scope of the new technology and the problems to be solved before it can be realized, and displayed the technical resources of the Federal laboratories and the ways by which entrepreneurs might avail themselves of them. The conference received top-level political attention: it was organized by Dr. William Graham, the President’s Science Advisor, and President Reagan himself gave a speech. The attendance was about 1500 people, including representatives of many Federal laboratories, universities, manufacturing companies, and a large number of consulting and market research companies.

The conference opened with a session in which Angelica Stacy (UC Berkeley), Paul Chu (Univ. Houston) and Robert Schrieffer (UC Santa Barbara) told the now-familiar story of the processing, structure, characteristics, and theory of the new ceramic superconductors. They were followed by Sadeg Faris (Hypres) and Sibley Burnett (GA Technology), who described their experiences in bringing products of the “old” superconductor technology to market: a fast sampling oscilloscope and magnets for a magnetic resonance imaging system, respectively. They had shown in their own ways that it really can be done.

President Reagan then gave the keynote address. He started by announcing some new progress in the nuclear arms negotiations in Geneva. Then he warmed to the subject of superconductivity, with apt quotations from Benjamin Franklin and Thomas Jefferson. He concluded by outlining the Administration’s Superconductivity Initiative. This includes modifications to the anti-trust laws, the patent laws, and the Freedom of Information Act, the establishment of four Superconductivity Research Centers (three at DoE laboratories and one at NBS-Boulder), and directions to various Federal scientific laboratories to increase their efforts in superconductivity. After his speech, the President toured the exhibits and the conference adjourned for lunch, at which Clayton Yeutter, the U.S. Trade Representative, gave a speech emphasizing the highly competitive nature of the potential international market and the formidable challenge from Japan.

The conference then turned to speculation about possible directions that the new superconductor technology might take. Theodore VanDuzer (UC Berkeley) gave a tutorial on the Josephson effect and other aspects of superconducting electronics. Fernand Bedard (NSA) then took up the theme of speculating about the future. He started from the notion that those who ignore history are condemned to repeat it, and reviewed the successes and failures of the past and the lessons that could be learned from them. He saw particular promise in a hybrid superconductor/semiconductor technology: a computer might combine the advantages of superconductor logic and transmission lines with the more established technology of semiconductor cache memory, for example.

Speculation on applications to transportation and electric power were then taken up by Craig Davis (Ford) and Narain Hingorani (EPRI), respectively. The two unfinished technologies in transportation are electric cars and magnetically levitated trains. Mr. Davis showed that even high temperature superconductors could not be expected to do much for electric cars, but felt there is more promise in magnetic levitation. In electric power systems, magnetic energy storage, underground transmission, and MHD generation all deserve careful consideration. To end the first day, Roland Schmitt (GE) and Mark Rochkind (Philips) reviewed activities in high temperature superconductor research in Japan and Western Europe.

The second day opened with descriptions of two superconductor systems that are already established as medical diagnostic tools and could derive quick benefit from a high temperature superconductor technology. Sam Williamson (NYU) described remarkable achievements of magnetoencephalography, at first in medical research and more recently in diagnosis. He suggested that in the future people may want routine brain checks as part of their physical examinations. Then John Stekley (IGC) described magnetic resonance imaging, with examples of what it can now do.

After this glimpse of what is possible, John Rowell (Bell Comm) and John Hulm (Westinghouse) reminded the audience of some of the major problems that must be solved before we can realize that possibility. For all the talk of Josephson junctions, microstrip transmission lines and hybrid superconductor/semiconductor circuits, YBCO films as we know them are incompatible with microcircuit processing as we know it. They have been successful only on SrTiO_3_ substrates, which are not compatible with semiconductor technology; they must be heat treated at 900°, which would destroy any nonrefractory layer in a circuit; and they are chemically very active, which will severely restrict the options for lithography. For large scale applications the present critical current density in the presence of even quite modest magnetic fields is too low by several orders of magnitude; the materials are too weak mechanically to sustain the stress from the Lorentz force; and the problem of electrodynamic stability is as yet completely unexplored. These problems are probably all soluble, but on a time scale of years, not months.

The Federal science laboratories are clearly a great national asset in getting a commercial superconductor technology launched. Eugene McAllister (White House) and Herman Postma (ORNC) discussed all the ways in which they are accessible to private companies. The desire of the Administration is to have private industry control the development of the new commercial technology, guided by market forces, and to use the resources of the Federal laboratories to the greatest possible extent.

The formal sessions of the conference closed with an address by John Herrington, Secretary of Energy. In the afternoon there were workshop sessions for informal discussion of the topics raised at the conference. There were sessions on industrial collaboration; access to Federal laboratories; finances, venture capital and small business; advances in the science of superconductivity; and industry/university/government cooperation. With these the conference ended on the optimistic note that the present administration considers the development of a commercial superconductor technology as being a test of the ability of the U.S. free-enterprise system to compete with other nations where the government plays a more active role in commercial affairs.

